# Up-regulation of circulating microRNA-17 is associated with lumbar radicular pain following disc herniation

**DOI:** 10.1186/s13075-019-1967-y

**Published:** 2019-08-13

**Authors:** Eivind Hasvik, Tiril Schjølberg, Daniel Pitz Jacobsen, Anne Julsrud Haugen, Lars Grøvle, Elina Iordanova Schistad, Johannes Gjerstad

**Affiliations:** 1 Department of Physical Medicine and Rehabilitation, Østfold Hospital Trust, Grålum, Norway; 2 Department of Work Psychology and Physiology, National Institute of Occupational Health, Oslo, Norway; 3 Department of Rheumatology, Østfold Hospital Trust, Grålum, Norway; 4 Department of Physical Medicine and Rehabilitation, Oslo University Hospital, Ullevål, Norway

**Keywords:** MicroRNA-17, Disc herniation, Nucleus pulposus, Lumbar radicular pain, Radiculopathy, Low back-related leg pain, Inflammation, TNF, Monocytes, Macrophages

## Abstract

**Background:**

Previous studies suggest that regulatory microRNAs (miRs) may modulate neuro-inflammatory processes. The purpose of the present study was to examine the role of miR-17 following intervertebral disc herniation.

**Methods:**

In a cohort of 97 patients with leg pain and disc herniation verified on MRI, we investigated the association between circulating miR-17 and leg pain intensity. A rat model was used to examine possible changes in miR-17 expression in nucleus pulposus (NP) associated with leak of NP tissue out of the herniated disc. The functional role of miR-17 was addressed by transfection of miR-17 into THP-1 cells (human monocyte cell line).

**Results:**

An association between the level of miR-17 in serum and the intensity of lumbar radicular pain was shown. Up-regulation of miR-17 in the rat NP tissue when applied onto spinal nerve roots and increased release of TNF following transfection of miR-17 into THP-1 cells were also observed. Hence, our data suggest that miR-17 may be involved in the pathophysiology underlying lumbar radicular pain after disc herniation.

**Conclusions:**

We conclude that miR-17 may be associated with the intensity of lumbar radicular pain after disc herniation, possibly through a TNF-driven pro-inflammatory mechanism.

**Electronic supplementary material:**

The online version of this article (10.1186/s13075-019-1967-y) contains supplementary material, which is available to authorized users.

## Introduction

Previous data show that lumbar intervertebral disc herniation may cause radicular leg pain [1]. In such pain conditions, mechanical pressure onto the nerve roots, but also the local inflammatory response following leak of nucleus pulposus (NP) tissue out of the immune-privileged disc, may be associated with the clinical symptoms [2–4]. In particular, radiating leg pain after disc herniation may have a clear neuro-inflammatory component induced by the contact between the immunogenic NP cells and the neighbouring tissue outside the disc. This involves activation of circulating monocytes and macrophages [5, 6], as well as release of TNF and other cytokines such as IL-1β and IL-6 [7, 8].

Previous studies suggest that microRNA (miR) may modulate neuropathic (e.g. [9, 10]) and inflammatory pain (e.g. [11, 12]). These miRs are small non-coding RNAs about 22 nucleotides in length that act by binding to the 3′UTR (untranslated region) of complementary target mRNA, which may either induce post-transcriptional silencing or mRNA degradation. Interestingly, miRs are extensively conserved across species and may therefore have many functions [13]. It is predicted that the activity of up to 50% of all protein-coding genes in mammals are controlled by miRs [14].

Several miRs have been found to affect expression of genes important for conditions related to the intervertebral discs [15–17]. Deregulated miR-155 and up-regulated miR-27a in the disc may be associated with apoptosis of human disc cells in vitro [18, 19], whereas up-regulation of miR-10b and miR-21 has been linked to abnormal NP cell proliferation and disc degeneration [20, 21]. Increased miR-146a expression may suppress inflammatory responses in the intervertebral disc induced by cytokines such as interleukin-1 [22]. In patients with pain after lumbar disc herniation, miR-223 may be associated with pain recovery [23].

It has been found that the miR-17-92 cluster, consisting of miR-17, miR-18a, miR-19a, miR-19b, miR-20a and miR-92a, may cooperatively regulate voltage-gated potassium channels and therefore affect development of neuropathic pain conditions [10]. Moreover, miR-17, miR-20a and miR-106a from the miR-17 family may regulate signal regulatory protein α (SIRPα) synthesis and SIRPα-mediated macrophage activation with increased TNF production [24]. Given the neuropathic and neuro-inflammatory nature of disc herniation-related leg pain, we hypothesized miR-17 to be involved in the pathophysiology of this condition. The aim of the present study was to examine the role of miR-17 with regard to mechanisms underlying leg pain intensity and inflammatory processes following disc herniation.

## Methods

### Clinical study

#### Setting and procedures

This study was part of a prospective, 1-year observational study on patients with lumbar radicular pain and disc herniation who were referred to the back clinic at Oslo University Hospital, Ullevål (OUH), Norway, during 2007 to 2009. Patients were invited to participate by the clinic staff. Inclusion criteria were age 18–60 years, low back-related leg pain with a corresponding lumbar disc herniation at the relevant side and level confirmed by magnetic resonance imaging and positive straight leg raise test (defined as radiating pain below the knee within 60° elevation). Exclusion criteria were lumbar spinal stenosis, previous disc surgery at the same level, lumbar spine fusion at any level, generalized musculoskeletal pain, inflammatory rheumatic disease, diabetic polyneuropathy, cardiovascular disease, cancer, psychiatric disease, neurological disease, alcohol or drug abuse, completion of another surgery within 1 month, pregnancy, non-European-Caucasian ethnicity or poor Norwegian language. Other invasive procedures such as epidural steroids and/or selective nerve root injections were not exclusion criteria. These treatments are not used regularly in Norway and were not part of the standard treatment regime in the present study (only selective nerve root blocks are used at OUH, mainly for diagnostic purposes).

Patients received a standard clinical examination and answered a comprehensive questionnaire at baseline, 6 weeks, 6 months and 1 year. Treatment followed practice as usual, without any pre-allocation or randomization. The conservative management consisted of a brief cognitive intervention, activity guidance during the acute phase and physiotherapy. The choice of additional surgical treatment was made at the discretion of each physician/surgeon. All participants received written information and signed an informed consent form. The study was approved by the Norwegian Regional Committee for Medical Research Ethics (South-East) and the Norwegian Social Science Data Services.

#### Patient-reported outcome measures

Pain severity was assessed by a numeric rating scale for leg pain with endpoints no pain (0) and worst possible pain (10). Duration of present pain prior to inclusion was recorded in weeks. Smoking status was recorded as smoker or non-smoker. Back pain-related disability was assessed using the Oswestry Disability Index (ODI) [25, 26] including 10 areas of pain and daily activities (pain intensity, personal hygiene, lifting, walking, sitting, standing, sleeping, sexual activity, social activity and travelling) with a total score range of 0–100. A higher score indicates greater disability. Anxiety and depression were assessed using the Hopkins Symptom Checklist-25 (HSCL-25) [27], a shortened version of the HSCL questionnaire [28], which includes 10 items to assess anxiety and 15 items to assess depression. Each item has four response categories ranging from not at all (1) to extremely (4), referring to symptoms during the previous week. The score is calculated as the mean of the completed items.

#### MicroRNA (miR) expression

Venous blood was collected and kept on ice for 45 min. After centrifugation at 2000×*g* and 4 °C and for 10 min, the supernatant serum was collected and stored in aliquots at − 80 °C until further analysis. Samples with visible haemolysis were excluded. Total small RNAs were extracted from 200 μL serum using the miRNeasy serum plasma isolation kit (Qiagen, Hilden, Germany) according to the manufacturer’s protocol. Synthetic *C*. *elegans* (C) miR-39-3p (Qiagen, Hilden, Germany) was spiked-in at a final concentration of 1.6 × 108 copies/μL after the initial denaturation, prior to extraction, to correct the extraction efficiency. The total RNA was eluted in 14 μL of RNase-free water. A fixed volume of 7 μL of eluate was used as input for the cDNA synthesis. RNA was converted to cDNA using the qScript™ microRNA cDNA synthesis kit (Quanta Biosciences, Gaithersburg, MD). qPCR was performed with PerfeCta® SYBR® Green Supermix on an Applied Biosystems (Foster City, CA) 7900 Real Time PCR System with the following conditions: 95 °C for 2 min, followed by 40 cycles at 95 °C for 5 s, 60 °C for 15 s and 70 °C for 15 s. MicroRNA expression levels were normalized to the mean of spiked-in miR C-miR-39 and presented as 2^−ΔCt^ values.

### Animal experiment

#### Surgery

Adult inbred female Lewis rats (*n* = 10, 165–206 g, Harlan Laboratories Inc., Bicester, UK) were sedated with isoflurane gas (Baxter International Inc., Deerfield, IL) and anaesthetized with 250 mg/ml urethane (1.8–2.7 g/kg body-weight i.p.) (Sigma-Aldrich Co., St. Louis, MO). Adequate surgical anaesthesia was verified by the absence of paw withdrawal and ear reflex to pinch. The rat core temperature was maintained at 36–37 °C by a feedback heating pad (homeothermic blanket control unit, Harvard Apparatus Ltd., Kent, UK).

A laminectomy with a lateral expansion on the left side was performed on vertebrae Th13–L1, corresponding to the spinal cord segments of L3–S1, where the sciatic nerve roots enter the spinal cord. The vertebral column was fixed with clamps, both rostral and caudal to the exposed spinal cord. The meninges were punctured by a cannula and carefully removed by two tweezers. We harvested NP tissue from 3 to 8 caudal intervertebral discs from a genetically identical donor rat (*n* = 10). The amount of graft harvested was enough to cover the dorsal nerve root with one of the bisected parts. One NP graft from each donor rat plus a control (made by the bisection) was used in the analyses. As previously described [29, 30], one piece was immediately frozen (native), and the other piece was applied onto the spinal dorsal nerve roots for 180 min before frozen (exposed). All animal experiments were approved by the Norwegian Animal Research Authority and were handled in accordance with the European Convention for the Protection of Vertebrate Animals used for Experimental and Other Scientific Purposes. The rats were euthanized immediately after the experiments.

#### MicroRNA-17 (miR-17) analysis

In accordance with the manufacturer’s protocol, total small RNA was isolated from frozen (− 80 °C) tissue using the miRNeasy micro kit (Qiagen, Hilden, Germany) and converted into cDNA using the miScript HiSpec reverse transcription buffer (Qiagen, Hilden, Germany). Then, 1 ng of cDNA was used in the qPCR reaction, performed on an Applied Biosystems (Foster City, CA) 7900 Real Time PCR System with the following conditions: 95 °C for 15 min, followed by 40 cycles at 95 °C for 5 s, 55 °C for 30 s and 70 °C for 30 s. The mean of the SNORD61 and SNORD68 reference genes was used for normalization of miR-17-5p expression. All primers were predesigned and delivered by Qiagen, Hilden, Germany. We performed no pooling of RNA after bisection of the NP grafts.

### TNF secretion induced by miR-17

#### Cultivation of THP-1 cells

THP-1 cells (a kind gift from Dr. Zienolddiny’s research group, Section of Toxicology, National Institute of Occupational Health, Oslo, Norway) were maintained in Gibco™ RPMI 1640 Medium with 10% foetal bovine serum (FBS) (Life Technologies, Grand Island, NY) and 1% HyClone™ Penicillin-Streptomycin (GE Healthcare Lifesciences, Logan, UT) at 37 °C and 5% CO_2_. The cell suspension was kept between 0.2 and 0.8 × 10^6^ cells/mL in T-75 TC flasks (Sarstedt, Nürnbrecht, Germany). The THP-1 cell line [31] is regarded as a reliable model to study monocyte and macrophage functions and has a response pattern similar to human peripheral blood mononuclear cell-derived monocytes and macrophages [32].

#### THP-1 cells exposed to miR-17

THP-1 cells were seeded as suspensions of 0.5 × 10^6^ cells/well in 1.7 mL RPMI 1640 with 10% FBS, in 6-well plates (Sarstedt, Nürnbrecht, Germany). We used Lipofectamine™ RNAiMAX Transfection Reagent (Invitrogen™, Life Technologies, Carlsbad, CA) to transfect THP-1 cells with 30 pmol of either MISSION® microRNA Mimic hsa-miR-17 (Sigma-Aldrich Co. LLC, MO) or mirVana™ miRNA Mimic, Negative Control#1 (Invitrogen™, Life Technologies, Carlsbad, CA). A transfection complex consisting of 1.25 μL Lipofectamine in 150 μL serum-free media plus 5 μL miR-17-5p was prepared and incubated at room temperature. As recommended by the manufacturer, 300 μL transfection complex was added to each well with cell culture and incubated at 37 °C with 5% CO_2_. After 48 h, cells were counted with NucleoCounter® NC-200™ (ChemoMetec, Allerod, Denmark) and separated from the surrounding medium by centrifugation at 135×*g* in 4 °C for 5 min. The conditioned medium was then collected, and the cell fraction was washed with PBS. Both medium and cells were stored at − 70 °C prior to analysis.

To demonstrate successful transfection of miR-17 into the THP-1 cells, total RNA (containing microRNA) in the cell fraction was purified through spin-columns with RNA/DNA Purification Kit (Norgen Biotek Corp, Thorold, ON, Canada) and converted into cDNA using qScript™ microRNA cDNA synthesis kit (Quanta Biosciences, Gaithersburg, MD). qPCR (QuantStudio5, Applied Biosystems, Härsling Industrial, Singapore) on hsa-miR-17-5p normalized to SNORD48, RNU6 and miR-24 was used to show uptake of miR-17 into the cells (Additional file 1: Figure S1). 1X PerfeCTa® SYBR® Green SuperMix with 300 nM miScript PerfeCTa® Universal PCR primer and PerfeCTa® microRNA assay (Quanta Biosciences, Gaithersburg, MD) for the miRs (10 μL RT-qPCR reactions) were used. qPCR was performed with the following conditions: 95 °C for 30 s, followed by 40 cycles of 95 °C for 5 s, 60 °C for 15 s and 70 °C for 10 s.

#### TNF analysis

Secretion of TNF (assayID: 171B5026M) in conditioned medium was analysed with Bio-Plex Pro™ cytokine assay (Bio-Rad Laboratories, Inc., Hercules, CA) on Bio-Plex MAGPIX (Luminex Corporation, Austin, TX) with Bio-Plex Manager™ MP Software (Bio-rad Laboratories, Inc., Hercules, CA) according to the instruction manual.

### Analyses and statistics

A linear mixed-effects regression model to control for dependency in the longitudinal data was used to examine the effect of miR-17 expression levels on leg pain intensity in patients. The main outcome variable was patient-reported leg pain (0–10) measured at baseline, 6 weeks, 6 months and 1 year. Variates associated with the outcome in prior research were included and consisted of age, sex, smoking (yes/no), surgical treatment after inclusion in the study (yes/no) and duration of present symptoms prior to inclusion [33]. The model random factors (random intercept for subject and random slope for time) were based on a best fit approach assessed by Akaike’s information criteria [34]. Unstructured covariance structure for the residuals was used. The model was assessed for adherence to linear mixed-effects regression assumptions. Robust estimates for the standard errors were used. No centring or imputation was performed. Collinearity was assessed by variance inflation factor (VIF) [35].

The difference in the rat miR expression between native and exposed NP tissue was analysed by the dependent-samples sign-test. The difference in TNF secretion by THP-1 cells transfected with scrambled miR (control) and transfected with miR-17 was analysed by the Wilcoxon rank sum test. Additionally, the percentage change from control condition to experimental condition was presented—calculated as change in location divided by the median of the control [36]. Effect size was estimated with the robust *probability of superiority* statistic, *A* [37].

#### Software

Analyses were done using Stata Statistical Software, version SE 15.1 (StataCorp LCC, College Station, TX, USA), R version 3.5.1 (R Core Team, R Foundation for Statistical Computing, Vienna, Austria) and RStudio version 1.1.463 (RStudio Team. RStudio Inc., Boston, MA, USA), with the packages *tidyverse* [38], *Lattice* [39] and *DescTools* [40]. The alpha level for all analyses was 0.05.

## Results

**Fig. 1:**
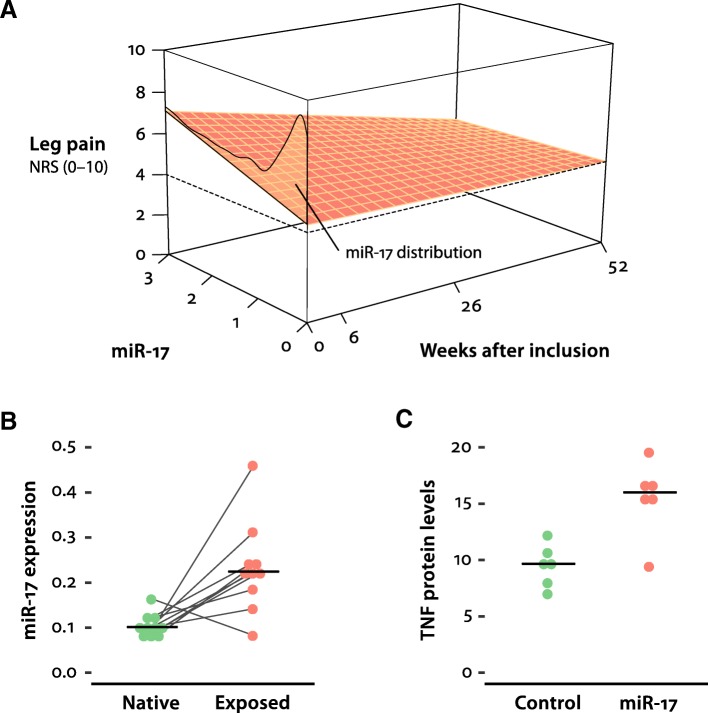
Role of miR-17 in disc herniation. **a** The interaction between miR-17 and time, with leg pain as outcome (clinical study). Increased levels of miR-17 are associated with higher leg pain intensity at baseline, levelling off towards 1 year. The density curve represents the distribution of miR-17 expression among patients. Upper range of miR expression restricted at ≤ 3. Estimates are based on the mixed-effects regression model. The other covariates in the model are held constant, using the most common category for surgery and smoker (non-surgery and non-smoker), median prior pain duration, female sex and mean age. **b** Expression levels (2^−ΔCt^ values) of miR-17 in native rat NP and rat NP exposed to spinal dorsal nerve roots, median 122% increase, *n* paired = 10, *p* = 0.022 (dependent-samples sign-test). **c** TNF protein levels (pg/mL) in the medium of THP-1 cells transfected with scrambled miR (control) or transfected with miR-17, median 66% increase, *n1* = *n2* = 12, *p* = 0.015 (Wilcoxon rank sum test)

In total, 97 patients were followed up for 1 year after disc herniation (Table 1). The estimates from the mixed-effects regression model demonstrated a positive relationship between leg pain intensity and miR-17 expression level at baseline, *B* = 0.862 (95% CI 0.348, 1.376), *Z* = 3.290, *p* = 0.001. A statistically significant interaction effect between miR-17 and time (week) was also observed, *B* = − 0.019 (− 0.034, − 0.004), *Z* = − 2.49, *p* = 0.013 (Table 2). The effect of miR-17 on leg pain gradually declined towards 1 year (Fig. 1a). The estimates for the subject-specific random factor (random intercept) was *B* = 0.7 (0.41–1.194). No collinearity was present (all VIF values < 3).

**Table 1:** Characteristics of included patients with lumbar radicular leg pain and disc herniation (*N* = 97)

	Baseline	1 year
Females, *n* (%)	58 (59.8)	
Age (years), mean (SD)	41.1 (9.8)	
Duration of current episode (weeks), median (IQR)	14 (8–24)	
Pain intensity (0–10), mean (SD)		
Low back pain	3.7 (2.7)	3.3 (3.1)
Leg pain*	5.7 (2.8)	2.8 (3.1)
Current smoker (baseline), *n* (%)	34 (35)	
Surgery during study period, *n* (%)		36 (37)
Selective nerve root blocks during study period, *n* (%)		2 (2)
Anxiety and depression (HSCL-25)^†^ (0–4), mean (SD)	1.74 (0.48)	1.51 (0.52)
Oswestry Disability Index (0–100), mean (SD)	36.1 (17.7)	19.3 (15.7)

*Only leg pain intensity was used for statistical analysis

^†^Hopkins Symptom Checklist-25

**Table 2:** Fixed effects parameter estimates from the linear mixed-effects regression model, with leg pain intensity during the study time span (1 year) as outcome (*N* = 97). No centring or standardization of coefficients. Robust SE estimates

	*B*	95% Confidence Interval		SE	*Z*	*p*
miR-17	0.862	0.348	1.375	0.262	3.290	0.001
miR-17 × time (week)	− 0.019	− 0.034	− 0.004	0.008	− 2.490	0.013
Time (week)	− 0.006	− 0.022	0.009	0.008	− 0.800	0.421
Surgery	− 2.701	− 3.470	− 1.931	0.393	− 6.880	< 0.001
Smoker	1.092	0.082	2.101	0.515	2.120	0.034
Prior duration (week)	0.017	0.001	0.032	0.008	2.130	0.033
Age	− 0.010	− 0.058	0.038	0.025	− 0.400	0.689
Sex (male)	0.151	− 0.852	1.153	0.512	0.290	0.768

To further investigate changes in miR-17 expression in NP cells following disc herniation, rat NP was applied onto the dorsal nerve roots. A substantial increase in the NP expression of miR-17 was observed in the NP exposed to neighbouring neuronal tissues. In fact, miR-17 expression in exposed NP tissue was 122% higher than the miR-17 expression in native NP tissue (median control 0.102; exposed 0.225), median difference 0.124 (97.9% CI 0.04, 0.208), *A* = 0.9 (95% CI 0.5, 1), dependent-samples sign-test, *S* = 9, *n paired* = 10, *p* = 0.022, two-sided (Fig. 1b).

Moreover, transfection of miR-17 into the THP-1 cells (human monocyte cell line) showed that miR-17 may regulate the release of TNF. Notably, the TNF level in the media of the THP-1 cells exposed to miR-17 was 66% higher than the TNF level in media of THP-1 cells exposed to scrambled miR (median control 9.668; miR-17 16.010), estimated difference in location 6.342 (95% CI 2.428, 9.544), *A* = 0.917 (95% CI 0.5, 1), Wilcoxon rank sum test, *W* = 3, *n1 = n2* = 12, *p* = 0.015, two-sided (Fig. 1c). Thus, an up-regulation of miR-17 in the NP tissue outside the immune-privileged disc and the local release of TNF from the monocytes induced by miR-17 were demonstrated.

## Discussion

Previous reports suggest that miRs may be involved in the regulation of pain in osteoarthrosis, rheumatoid arthritis and complex regional pain syndrome [9, 11, 12]**.** In the present study, we extend these observations and show that high miR-17 levels in serum of patients with disc herniation are associated with increased radicular pain. Moreover, our findings suggest that up-regulation of miR-17 in NP may facilitate the local release of TNF from circulating immune cells, for example monocytes (primitive macrophages). Hence, miR-17 may influence leg pain intensity after disc herniation, potentially through a TNF-driven pro-inflammatory mechanism.

The role of miR-17 has previously been addressed in several immune-mediated diseases [41, 42]. Also, earlier experimental studies show that the miR-17 family and miR-17-92 cluster may be important in the activation of macrophages, T cell function as well as cytokine production in B cells [24, 43–45]. Moreover, previous observations show increased counts of macrophages, as well as T and B cells, in disc herniations [46, 47]. However, a direct relationship between the activation of the immune cells, the inflammatory process and clinical symptoms has not been established [48–50].

Most patients experience a substantial recovery during the first 3 months following disc herniation [51]. During this period, the disc material can be reabsorbed. It is possible that the recruitment of pro-inflammatory cells reflects a process of resorption of disc material and that the resolution of pain over time correlates with macrophage presence [6, 52]. This process could also, if miR-17 controls the release of TNF, be influenced by miR-17. However, protrusions (when the outer annular lamellae remain intact) or extrusions (when the annular lamellae are ruptured with leakage of NP) may be associated with different inflammatory processes and symptoms [1, 53–55].

Previous in vitro experiments suggest that the inflammatory response of macrophages may be mediated through miR-17 targeting SIRPα, which in turn lead to increased expression of TNF [24]. In the present study, we demonstrated an up-regulation of miR-17 when the NP tissue leaks out of the disc, but also provided data suggesting a possible local TNF-driven pro-inflammatory effect on relevant circulating immune cells. Such immune cells (monocytes or macrophages) may infiltrate herniated discs in patients [5, 6]. Thus, our results emphasize the clinical relevance of the link between miR-17 and TNF-driven inflammation.

TNF is regarded as a key cytokine associated with disc herniation and neuropathic pain [56, 57] and also produces histologic changes similar to those seen in nerve roots exposed to NP [58, 59]. Moreover, in disc herniation patients, the level of local TNF correlates well with inflammation and pain intensity [46, 57, 60]. After disc herniation, TNF may also promote natural resolution of disc herniation through matrix metalloproteinase-3 (MMP-3) [61]. Thus, TNF may act in a transient or time-restricted manner. Although this direct effect may be short lasting, TNF can still initiate the up-regulation of downstream mediators leading to neuropathic pain [62, 63].

Previous data show that TNF may be a potential target of miR-17 in several cell types [64, 65]. There is some evidence that downregulation of miR-17, together with miR-20a and miR-106a, may be involved in promoting differentiation and maturation of monocytic cells, suggesting possible cross-talk effects or feedback loops [66]. Only a few nucleotides differ between miR-20a and miR-17, and these differences reside outside the seed region [43]. Moreover, miR-106b is a homologue of miR-17 and belongs to one of two miR-17-92 cluster paralogs miR-106a-363 and miR-106b-25. Overlapping functions between these miRs are therefore likely [67].

At least in animals, several other miRs are co-expressed with miR-17 and may be associated with neuropathic pain [10, 24, 68]. Thus, the observed association between miR-17 and pain in the present study may be explained by more complex mechanisms, such as regulation of voltage-gated potassium channels [10]. Moreover, it has been argued that the miR-17-92 cluster may be important for nervous system regeneration by promoting neurogenesis, angiogenesis and axonal outgrowth in embryonic cortical neurons [69, 70]. Thus, this cluster could be involved in neural regeneration following nerve injury initiated by the disc herniation, and indirectly be linked to the increase in pain intensity.

The present data point to physiological changes in serum, i.e. increased circulating miR-17, which may be associated with pain after disc herniation. Moreover, previous data from our group have revealed that persistent pain after disc herniation may be associated with increased circulating cytokine levels and changes in protein profiles [71, 72]. However, since these studies are based on analyses of serum (not NP tissue), the influence of socioeconomic status, lifestyle or other factors not directly related to disc herniation, may also complicate the interpretations of the results. Still, the estimated regression coefficient of our data showed a clinically relevant association between miR-17 and pain. Moreover, our in vivo and in vitro experiments indicate a local NP up-regulation of miR-17 after disc herniation that may facilitate the release of TNF (and probably other cytokines) from circulating immune cells. Thus, our data support the hypothesis that miR-17 may be involved in the pathophysiology of lumbar radicular pain. In the present study, we choose to focus on the effect of miR-17 on immune cells, that is, inflammatory processes. Whether this is relevant for the expression and function of miR-17 in the dorsal nerve roots, nociceptive pathways or the brain, remains to be investigated.

## Conclusion

The results from the present study showed that miR-17 expression may be associated with increased leg pain intensity in patients following disc herniation. Up-regulation of miR-17 in the rat NP tissue when applied to spinal nerve roots and increased release of TNF following transfection of miR-17 into the THP-1 cells were also observed. We conclude that miR-17 may be associated with the intensity of lumbar radicular leg pain after disc herniation, possibly through a TNF-driven pro-inflammatory mechanism.

## Additional file

Additional file 1: **Figure S1.** Transfection of miR-17 into the THP-1 cells. MiR-17-5p normalized to SNORD48, RNU6 and miR-24 was used to show uptake of miR-17 into the THP-1 cells. (A) Expression levels for the different experimental conditions, normalized to control. (B) Corresponding cell viability measures. (PDF 40 kb)

## Data Availability

The datasets used and analysed during the current study are available from the corresponding author on reasonable request.

## Abbreviations

FBS: Foetal bovine serum
HSCL-25: Hopkins Symptom Checklist-25
IL: Interleukin
miR: MicroRNA
MMP: Matrix metalloproteinase
NP: Nucleus pulposus
ODI: Oswestry Disability Index
OUH: Oslo University Hospital
SIRPα: Signal regulatory protein α
TNF: Tumour necrosis factor
VIF: Variance inflation factor
