# Supplementary material for: Up-regulation of circulating microRNA-17 is associated with lumbar radicular pain following disc herniation
Source: Arthritis Res Ther. 2019 Aug 13;21:186. doi: 10.1186/s13075-019-1967-y (PMC6693234; doi:10.1186/s13075-019-1967-y)
Supplement: Supplementary file 1 — Figure S1. Transfection of miR-17 into the THP-1 cells. MiR-17-5p normalized to SNORD48, RNU6 and miR-24 was used to show uptake of miR-17 into the THP-1 cells. (A) Expression levels for the different experimental conditions, normalized to control. (B) Corresponding cell viability measures. (PDF 40 kb) [file 13075_2019_1967_MOESM1_ESM.pdf]

Supplementary material

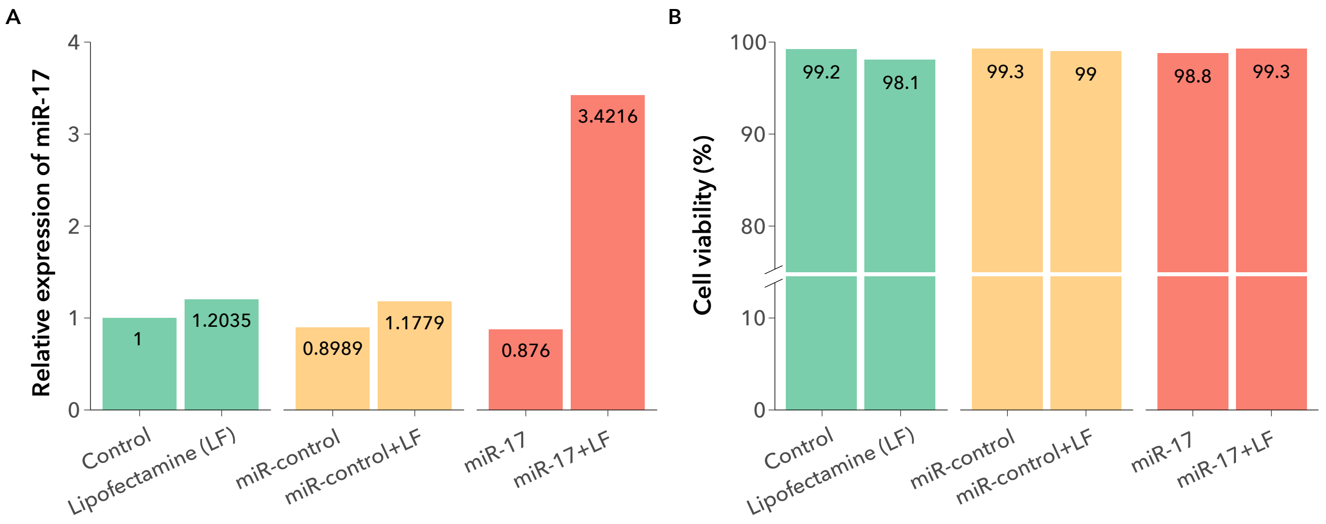

Supplementary Figure 1. Transfection of miR-17 into the THP-1 cells. MiR-17-5p normalized to SNORD48, RNU6 and miR-24 was used to show uptake of miR-17 into the THP-1 cells. **(A)** Expression levels for the different experimental conditions, normalized to control. **(B)** Corresponding cell viability measures.
